# Wasting resources in health care – unnecessary hospitalisations and over-medicalisation: The health workers’ or the health systems’ fault? Evidence from Romania and Tajikistan and implications for global health

**DOI:** 10.7189/jogh.13.03018

**Published:** 2023-05-05

**Authors:** Sophie Jullien, Susanne Carai

**Affiliations:** 1 World Health Organization Regional Office for Europe, Quality of care and patient safety office, Athens, Greece; 2 World Health Organization Regional Office for Europe, Child and Adolescent Health, Copenhagen, Denmark; 3 Witten Herdecke University, Witten, Germany

Quality of care is a key element of universal health coverage [1]. The World Health Organization (WHO) defines it as the degree to which health services increase the likelihood of desired health outcomes; health care should be effective, safe, people-centred, timely, equitable, integrated, and efficient [1,2]. Waste in health care is any activity that does not add value to patient care and should be avoided [2]. Hospitals have a long-standing major role in patient survival. However, hospitalisation can cause psychological, emotional, and physical distress, increase the risk of nosocomial infections, contribute to the disruption of work and education, and represent a considerable financial burden incurred both by patients and the health system [3]. In the WHO European Region, 10% of patients experience preventable harm or adverse events in hospitals [4]. Hospitalisation should therefore be limited to those who cannot be safely managed at the primary health care (PHC) level.

Health system evaluations were conducted in Tajikistan and Romania in 2021 to quantify unnecessary and unnecessarily prolonged hospitalisations in children and pregnant women. The methodology (identical for both countries) and detailed findings are described elsewhere [5,6]; we summarize the main findings in **Table 1**. Unnecessary and unnecessarily prolonged hospitalisations of children, pregnant women, and women hospitalised for delivery were common, along with the misuse of antibiotics and prescription of multiple unnecessary and invasive drugs. Additional observations from Romania revealed the abuse of ambulance services by patients with non-severe conditions, routine separation of newborns from their mothers just after birth, and delayed start of and lack of support for breastfeeding.

**Table 1:** Main findings of health systems evaluation in Tajikistan and Romania, 2021

		Tajikistan	Romania
**Number of participants**	Children	440	209
	Pregnant women	422	349
	Women hospitalized for delivery	-	240
**Unnecessary hospitalisations**	Children*	40.5%	57.9%
	Pregnant women†	69.2%	56.2%
**Unnecessarily prolonged hospitalisations**	Children*	63.0%	44.4%
	Pregnant women†	39.2%	23.3%
	Women hospitalised for delivery‡	-	45.8%
**Median duration of hospitalisation**	Children*	8 d	4 d
	Pregnant women†	7 d	2 d
**Antibiotics during hospitalisation**	Children, overall*	92.5%	66.0%
	Children with diarrhoea	85.9%	53.1%
	Pregnant women†	28.9%	30.1%
**Use of ambulance**	Children*	-	22.9%

d – days

*Children aged 2-59 mo hospitalised with a primary diagnosis of an acute respiratory infection or acute diarrhoea.

†Pregnant women hospitalised with a primary diagnosis of threatened premature labour, threatened abortion, premature ruptures of membranes, or mild to moderate pre-eclampsia.

‡Women with term pregnancy hospitalised for delivery.

Besides these quantitative data being essential to monitor progress towards improving quality of care, we reflect here on their cause and impact within the health systems. These findings result from health worker practices and health care facility organisation and are determined by, and reflective of, major issues with the health systems’ structure and functions.

Despite PHC being recognized as key to achieving universal health coverage, PHC facilities remain largely underfinanced and underutilized globally, including in Tajikistan and Romania [7,8]. In these two countries, the population’s perception that hospital care is superior to that provided at PHC level contributes to the high prevalence of unnecessary hospitalisations. Strengthening PHC by improving both its infrastructure and the health worker’s capacity is fundamental to avoiding harm and reducing waste of resources. Increased awareness on the benefits of home treatment and adequate follow-up at PHC over hospitalisation would also contribute to shifting care seeking from hospitals to PHC facilities.

Second, financing mechanisms for health care services significantly affect the quality and outcomes of care [2]. In Tajikistan and Romania, a small portion of public funds is allocated to the health sector (absolute terms and in proportion to GDP). This is reflected in low salaries of health workers in the public sector and high out-of-pocket payments (both formal and informal) for health care [9]. Reliance on informal payments, alongside budgets and salaries linked to hospital bed occupancy (the case for Tajikistan), incentivises unnecessary hospitalisations and are likely leads to non-evidence-based treatment and prescribing practices and supplier-induced demand for caesarean sections (the case for Romania). In Romania, allocation of public resources to the health sector has improved since 2015 but the changes of behavioural practices lag behind. In 2019, out-of-pockets payments were still above the EU average [8]. Health systems relying on out-of-pocket payments lead to poor health outcomes and increased inequalities; funding should be based on mandatory insurance schemes [10]. Reviewing funding and financing systems regarding incentives for unnecessary hospitalisation should be a priority for both countries and beyond, to allow health systems to reduce waste, improve quality of care, and move towards universal health coverage.

Third, health care workers’ competencies and knowledge for providing quality care depends on the quality of the pre-service education and in-service specialization they received. WHO recommends updating university curricula to include latest evidence-based standards and integrate principles of quality and quality improvement into pre-service education [2]. This remains an unfinished agenda for many countries, including Tajikistan and Romania. Specialist accreditation requirements are not comparable across countries. Tajikistan and Romania lose many graduates (medical and nursing staff) to health systems offering better working conditions [8]. University curricula and specialist accreditation requirements in both countries should be reviewed and revised. Benchmarking against other countries is recommended.

**Figure:**
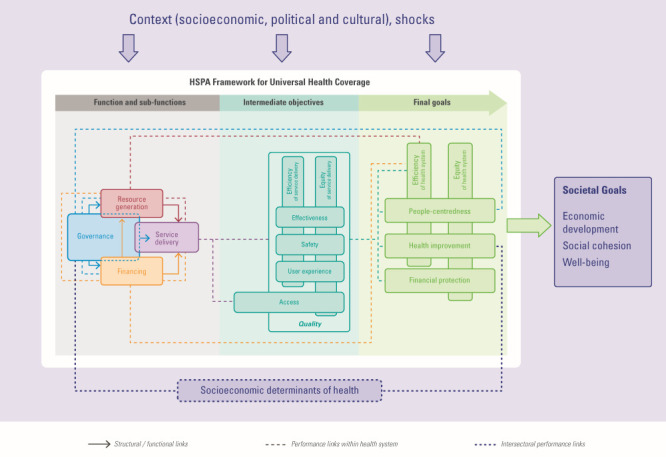
Photo: Health system performance assessment (HSPA) framework for universal health coverage. Source: Extracted from [15], free to use under CC BY-NC-SA 3.0 IGO license.

Fourth, the adequate utilization of trained midwives is key to delivering quality of care to mothers and newborns, yet their role remains undervalued in many countries, including Tajikistan and Romania [11]. In the former, midwives are not allowed to practice the full scale of their skills despite the promising establishment of the Association of Midwives of the Republic of Tajikistan [12]. In the later, there are 1.6 midwives per 10 000 population, much below the WHO regional average of 4.1 per 10 000 [13]. All countries should recognise the essential role of midwives for maternal, newborn, sexual, and reproductive health, and clearly define their role and responsibilities. This will contribute to reducing unnecessary hospitalisations and medicalisation of care such as unnecessary caesarean sections (the case in Romania), while ensuring evidence-based routine care of healthy mothers and their babies, such as skin-to-skin contact after delivery and breastfeeding support.

Changes in health systems cannot be carried out successfully without considering health care workers and communities; beliefs and barriers to evidence-based practices need to be addressed within the cultural context. Raising awareness and improving health literacy in the Tajik and Romanian communities is needed: patients and caregivers often do not feel adequately cared for if no medication is prescribed by their doctors, pressuring them into prescribing antibiotics. Communities and parents must be informed about the self-limiting nature of many childhood diseases, the harm and side-effects of antibiotics, and their ineffectiveness against viral infections. Also, patients often consider hospital care as superior to PHC and little is known or recognised about the costs, risks, and potential harms of unnecessary hospitalisations and treatments.

Understanding communities’ and health workers’ behaviours, practices, and perceptions is thus crucial for informing how to organize well-performing health systems and reduce waste in Tajikistan and Romania and beyond [14]. We fully recognize that in the current health system of the two countries that were assessed, hospitalisation may provide relieve to women and families with low resources or for other social reasons. Following this health system evaluation, the Ministry of Health and Social Protection of the Population in Tajikistan has committed to carry out further work to understand the root causes of unnecessary hospitalisations and polypharmacy and to engage in a participatory policy dialogue.

These health system evaluations were conducted employing a robust methodology; despite limitations inherent to retrospective data collection from medical records, it allows the measurement of indicators highly relevant for monitoring health system performance at different time points. It can be used in diverse settings, as shown by its application in high- and low-income countries, where it could likewise reveal waste of resources and potential harm. Unnecessary and unnecessarily prolonged hospitalisations are directly linked to quality of care and may be used for measuring effectiveness, safety, and access within the health system performance assessment framework for universal health coverage [15]. This new framework for policy analysis facilitates the identification of possible origins and impact of poor performance on a particular health system outcome, essential for policymakers identifying ways to strengthen the health system.

The findings of these health system evaluations and understanding the rationale behind the barriers to provision of quality of care and high health systems’ performance will be insightful for other countries committed to achieving universal health coverage with high quality health services that minimize waste.

“Without quality, universal health coverage remains an empty promise” [16]. Quality is not a given – actions are clearly required.
